# Trends in heart disease mortality among breast cancer survivors in the US, 1975–2017

**DOI:** 10.1007/s10549-022-06515-5

**Published:** 2022-02-02

**Authors:** Jacqueline B. Vo, Cody Ramin, Ana Barac, Amy Berrington de Gonzalez, Lene Veiga

**Affiliations:** 1 Radiation Epidemiology Branch, Division of Cancer Epidemiology and Genetics, National Cancer Institute, Bethesda, MD USA; 2 Director of Cardio-Oncology and Professor of Medicine, Medstar Heart and Vascular Institute, Georgetown University, Washington, DC USA; 3 Present Address: Cancer Prevention Fellowship Program, Division of Cancer Prevention, Bethesda, MD USA

**Keywords:** Breast cancer, Cancer survivorship, Heart disease mortality, Descriptive epidemiology

## Abstract

**Purpose:**

Heart disease is a significant concern among breast cancer survivors, in part due to cardiotoxic treatments including chemotherapy and radiotherapy. Long-term trends in heart disease mortality have not been well characterized. We examined heart disease mortality trends among US breast cancer survivors by treatment type.

**Methods:**

We included first primary invasive breast cancer survivors diagnosed between 1975 and 2016 (aged 18–84; survived 12 + months; received initial chemotherapy, radiotherapy, or surgery) in the SEER-9 Database. Standardized mortality ratios (SMRs) and 10-year cumulative heart disease mortality estimates accounting for competing events were calculated by calendar year of diagnosis and initial treatment regimen. *P*_trends_ were assessed using Poisson regression. All statistical tests were 2-sided.

**Results:**

Of 516,916 breast cancer survivors, 40,812 died of heart disease through 2017. Heart disease SMRs declined overall from 1975–1979 to 2010–2016 (SMR 1.01 [95%CI: 0.98, 1.03] to 0.74 [0.69, 0.79], *p*_trend_ < 0.001). This decline was also observed for survivors treated with radiotherapy alone and chemotherapy plus radiotherapy. A sharper decline in heart disease SMRs was observed from 1975 to 1989 for left-sided radiotherapy, compared to right-sided. In contrast, there was a non-significant increasing trend in SMRs for chemotherapy alone, and significant by regional stage (*p*_trend_ = 0.036). Largest declines in 10-year cumulative mortality were observed from 1975–1984 to 2005–2016 among surgery only: 7.02% (95%CI: 6.80%, 7.23%) to 4.68% (95%CI: 4.39%, 4.99%) and radiotherapy alone: 6.35% (95%CI: 5.95%, 6.77%) to 2.94% (95%CI: 2.73%, 3.16%).

**Conclusions:**

We observed declining heart disease mortality trends by most treatment types yet increasing for regional stage patients treated with chemotherapy alone, highlighting a need for additional studies with detailed treatment data and cardiovascular management throughout cancer survivorship.

**Supplementary Information:**

The online version contains supplementary material available at 10.1007/s10549-022-06515-5.

## Background

Advances in cancer treatment have contributed to the 5-year survival rates among breast cancer survivors approaching 90% [1]. These advances have shifted attention to the late adverse effects of treatment. Cardiovascular disease is now the leading cause of non-cancer deaths in women diagnosed with breast cancer in the US [2, 3]. Chemotherapy and radiotherapy both increase the risk of cardiovascular disease, and these adverse effects can occur acutely during treatment or decades after treatment has ended. Among older breast cancer survivors, the risk of dying from heart disease is now greater than breast cancer itself [2].

Etiology of cardiotoxicity varies by cancer treatment type and time since diagnosis. Anthracyclines and trastuzumab are associated with the risk of cardiomyopathy and heart failure, and the risk is significantly higher when used together [4–7]. Heart failure may present after anthracycline use, when ventricular dilation and dysfunction may be irreversible [7]. Limited data are available on the long-term cardiotoxic effects of trastuzumab; however, in the absence of anthracycline therapy, its cardiac effects are believed to be largely reversible after cessation of treatment [7]. Radiotherapy is associated with coronary artery disease and appears approximately 5–20 years after treatment completion [7–10].

Increased understanding of treatment-related adverse effects for breast cancer survivors has contributed to declines in anthracycline use [11], selection of alternative, less cardiotoxic treatments with similar effectiveness in certain breast cancers [12, 13], as well as implementation of modern radiotherapy techniques with reduced radiation exposure to the heart [14]. These adaptations in cancer treatment and increased awareness of cardiotoxicity should have impacted heart disease mortality rates among breast cancer survivors. Yet, potential shifts in heart disease mortality among US breast cancer survivors by treatment type over time have not been well characterized. In this study, we examined the long-term trends in heart disease mortality among women diagnosed with a first primary invasive breast cancer in the US using data from the Surveillance, Epidemiology, and End Results (SEER)-9 cancer registries according to initial treatment regimen (chemotherapy, radiotherapy, or surgery) and breast cancer characteristics from 1975 to 2017.

## Methods

### Data source

This retrospective, population-based cohort study utilized the SEER-9 database for breast cancer survivors, and reference cohort from the US Mortality data. SEER-9 registries cover 9 registries throughout the USA and represent approximately 9.4% of the US population and leverages cancer registry data to characterize cancer incidence, treatment, and survival. US Mortality Data are maintained by the National Center for Health Statistics (NCHS) of the Centers for Disease Control and Prevention (CDC). Causes of death and population data were ascertained from death certificates. This research was exempt of Institutional Review Board by the National Institutes of Health Office of Human Subjects Research based on the usage of deidentified existing data.

### Study population

We studied long-term patterns of heart disease mortality among 516,916 women diagnosed with a first primary, invasive breast cancer between ages 18–84, survived 12 months or longer, and received initial surgery, chemotherapy, or radiotherapy within SEER-9. We included first primary breast cancer only since treatment information in SEER is limited to first course of treatment. Women were diagnosed with breast cancer between January 1, 1975 and December 31, 2016 with follow-up until December 31, 2017.

### Heart disease mortality

Our outcome of interest was heart disease mortality identified using consistent site groupings over time by the International Classification of Diseases (ICD)-8 (1976–1978) and ICD-9 (1979–1998): 390–398, 402, 404, 410–429; and ICD-10 codes (1999–2017): I00-I09, I11, I13, I20-I51. Heart disease mortality was ascertained since this is a clinically relevant outcome for the selected treatments and the breast cancer population [7, 15].

### Treatment information

We classified women according to their initial breast cancer treatment: *surgery only* (women who received surgery but no/unknown history of chemotherapy or radiotherapy; *n* = 197,449), *chemotherapy alone* (women who received chemotherapy, with or without surgery, but no/unknown history of radiotherapy; *n* = 76,479), *chemotherapy plus radiotherapy* (women who received both chemotherapy and radiotherapy, with or without surgery; *n* = 102,838), and *radiotherapy alone* (women who received radiotherapy, with or without surgery, but no/unknown history of chemotherapy; *n* = 140,150). The treatment groups were mutually exclusive. Further, we present a subgroup analysis for radiotherapy alone by laterality of the breast cancer (left-sided and right-sided).

In the SEER database, trastuzumab was included under chemotherapy until 2013 when it was classified under biologic therapy/immunotherapy [16]. The SEER database does not provide detailed information on specific drugs, and therefore, we were unable to distinguish trastuzumab from other biologic therapy/immunotherapy agents from 2013 onward. To remain consistent, we considered trastuzumab under the chemotherapy treatment group for the entire study period and conducted a sensitivity analysis by including the biologic therapy/immunotherapy category in the chemotherapy group for diagnoses after 2013.

### Statistical analyses

Patients were followed beginning 1 year after their first primary breast cancer diagnosis (to estimate treatment completion) until date of last contact, death, or end up study period (December 31, 2017), whichever came first. To investigate heart disease risk relative to general population, we calculated standardized mortality ratios (SMRs) and corresponding 95% confidence intervals (CIs) comparing risk of heart disease deaths from the breast cancer cohort to the US general female population. SMRs were calculated by dividing the *observed* number of heart disease deaths among breast cancer survivors by the *expected* number in the US female general population, adjusted for age at death (5-year groups), race (White/Black/other), and year of death. We examined trends in heart disease SMRs by calendar year of breast cancer diagnosis and treatment type (surgery only/chemotherapy alone/chemotherapy plus radiotherapy/radiotherapy alone), latency defined as time since breast cancer diagnosis (1–9 years/10–19 years/20 + years), age of breast cancer diagnosis (18–49 years/50–59 years/60–69 years/70–84 years), and breast cancer stage (localized/regional/distant) (described further in Supplement A).

Tests for trend of SMRs by calendar year of breast cancer diagnosis (continuous) were assessed using Poisson regression model, with expected events as the offset. A sensitivity analysis was conducted to examine the SMRs by censoring follow-up at a second invasive cancer diagnosis, as development of a second cancer could increase risk of mortality. To assess the clinical burden of heart disease mortality, we calculated cumulative mortality (and corresponding 95% CIs) from heart disease, taking into account the competing risk of death from other causes [17]. We examined 10-year cumulative mortality estimates by calendar year of breast cancer diagnosis, stratified by treatment type. SMRs were conducted using SEER*Stat version 8.3.8. Cumulative mortality (using the *stcompet* package) and *p*_trend_ tests were conducted in Stata version 16 (StataCorp, College Station, TX). All tests were 2-sided with statistical significance set at *p* < 0.05.

## Results

### Descriptive characteristics

From 1975 to 2016, there were 516,916 one-year breast cancer survivors with a median follow-up time was 9.59 years (interquartile range of 4.58–16.84). Of these survivors, 38.20% received surgery only; 14.80% received chemotherapy alone; 19.89% received chemotherapy plus radiotherapy; and 27.11% received radiotherapy alone as their initial treatment (Table 1). The majority of localized cancers received either surgery only (44.08%) or radiotherapy alone (35.26%); regional cancers mostly received surgery only (29.48%), chemotherapy alone (23.72%), or chemotherapy plus radiotherapy (33.53%); and distant cancers mostly received chemotherapy alone (34.14%) or chemotherapy plus radiotherapy (35.18%). Survivors who received surgery only or radiotherapy alone were older at breast cancer diagnosis than those who received chemotherapy alone or chemotherapy plus radiotherapy. Treatment patterns were broadly similar by race. Use of surgery only declined over time from 1975–1979 to 2010–2016 (69.19% to 21.46%), whereas chemotherapy plus radiotherapy use (4.07% to 28.71%) and radiotherapy alone (17.74% to 34.19%) increased over time. Overall, there were 246,205 deaths, and 40,812 (16.58%) died of heart disease. A large proportion of survivors who died of heart disease had received surgery only (50.35%), followed by radiotherapy alone (27.91%).

**Table 1:** Overall characteristics of breast cancer survivors, 1975–2016

	Overall (*N* = 516,916; 100%)		Surgery only (*n* = 197,449; 38.20%)		Chemotherapy alone (*n* = 76,479; 14.80%)		Chemotherapy plus radiotherapy (*n* = 102,838; 19.89%)		Radiotherapy alone (*n* = 140,150; 27.11%)	
	*n*	%	*n*	%	*n*	%	*n*	%	*n*	%
Surgery										
Surgery	504,821	100	197,449	39.11	70,595	13.98	99,746	19.76	137,031	27.14
No Surgery	12,095	100	0	0.00	5884	48.65	3092	25.56	3119	25.79
Follow up time in years [mean (SD)]	11.65 (8.63)		13.26 (9.61)		10.63 (8.43)		9.85 (7.01)		11.27 (7.94)	
Median (IQR)	9.59 (4.58–16.84)		10.96 (5.25–19.41)		8.29 (3.75–15.63)		8.33 (3.96–14.46)		9.55 (4.71–16.29)	
Stage										
Localized	321,659	100	141,790	44.08	28,160	8.75	38,290	11.90	113,419	35.26
Regional	167,904	100	49,495	29.48	39,827	23.72	56,294	33.53	22,288	13.27
Distant	21,178	100	3194	15.08	7231	34.14	7451	35.18	3302	15.59
Missing	6175	100	2970	48.10	1261	20.42	803	13.00	1141	18.48
Laterality										
Left	262,367	100	100,614	38.35	38,806	14.79	52,186	19.89	70,761	26.97
Right	253,110	100	96,298	38.05	37,243	14.71	50,450	19.93	69,119	27.31
Bilateral	381	100	166	43.57	100	26.25	47	12.34	68	17.85
Unknown	1058	100	371	35.07	330	31.19	155	14.65	202	19.09
Age at cancer diagnosis										
Mean (SD)	59.03y (13.03y)		62.45y (13.16y)		52.73y (11.81y)		52.81y (11.29y)		62.20y (11.67y)	
18–49 years	134,164	100	37,748	28.14	32,337	24.10	42,045	31.34	22,034	16.42
50–59 years	127,715	100	40,467	31.69	22,045	17.26	31,717	24.83	33,486	26.22
60–69 years	129,099	100	50,304	38.97	15,081	11.68	20,923	16.21	42,791	33.15
70–84 years	125,938	100	68,930	54.73	7016	5.57	8153	6.47	41,839	33.22
Race										
White	430,953	100	170,256	39.51	61,231	14.21	80,501	18.68	118,965	27.61
Black	45,751	100	14,884	32.53	8670	18.95	12,085	26.41	10,112	22.10
Other	40,212	100	12,309	30.61	6578	16.36	10,252	25.49	11,073	27.54
Calendar year of breast cancer diagnosis										
1975–1979	37,418	100	25,888	69.19	3371	9.01	1522	4.07	6637	17.74
1980–1984	42,885	100	27,725	64.65	6018	14.03	2307	5.38	6835	15.94
1985–1989	54,885	100	33,059	60.23	6774	12.34	3769	6.87	11,283	20.56
1990–1994	60,508	100	28,507	47.11	9223	15.24	7452	12.32	15,326	25.33
1995–1999	68,466	100	23,337	34.09	10,048	14.68	14,497	21.17	20,584	30.06
2000–2004	71,206	100	18,828	26.44	11,064	15.54	20,615	28.95	20,699	29.07
2005–2009	71,611	100	16,517	23.06	12,780	17.85	21,114	29.48	21,200	29.60
2010–2016	109,937	100	23,588	21.46	17,201	15.65	31,562	28.71	37,586	34.19
Causes of death										
All cause	246,205	100	125,070	50.80	34,067	13.84	31,538	12.81	55,530	22.55
Breast cancer	98,524	100	37,152	37.71	23,043	23.39	21,466	21.79	16,863	17.12
Heart disease	40,812	100	25,973	50.35	2475	10.70	2039	11.05	10,325	27.91

Treatments were grouped by surgery only (and no known history of chemotherapy or radiotherapy), chemotherapy alone (± surgery and no known history of radiotherapy), chemotherapy plus radiotherapy (± surgery), and radiotherapy alone (± surgery and no known history of chemotherapy)

*Y* years, *SD* standard deviation, *IQR* interquartile range

### Heart disease SMRs

#### By treatment type

Overall, heart disease mortality in breast cancer survivors declined significantly relative to the general population by the calendar year of breast cancer diagnosis, from an SMR of 1.01 (95%CI: 0.98, 1.03) in 1975–1979 to SMR of 0.74 (95%CI: 0.69, 0.79) in 2010–2016, *p*_trend_ < 0.001 (Table 2). This declining trend in SMRs was also observed for patients treated with chemotherapy plus radiotherapy and radiotherapy alone. Left-sided radiotherapy had a greater decline in the SMRs for heart disease in the earlier years, from 1.77 (95%CI: 1.64,1.91) in 1975–1979 to 0.93 (95%CI: 0.87,0.99) in 1985–1989, compared to right-sided (SMR 1.39 [95%CI: 1.27,1.51] to SMR 0.84 [95%CI: 0.78,0.90] for same time period), while the decline in SMRs between these two groups after 1990 was similar. In contrast, there was a non-significant increasing trend for heart disease mortality among survivors treated with chemotherapy alone, with an SMR of 0.88 (95%CI: 0.77, 0.99) in 1975–1979 that increased to 1.01 (95%CI: 0.81, 1.25) in 2010–2016, *p*_trend_ = 0.11.

**Table 2:** Heart disease SMRs by calendar year of diagnosis and treatment type

	Overall (*N* = 516,916)			Surgery only (*n* = 197,449)			Chemotherapy alone (*n* = 76,479)			Chemotherapy plus radiotherapy (*n* = 102,838)		
	O	SMR	95% CI	O	SMR	95% CI	O	SMR	95% CI	O	SMR	95% CI
Calendar year of diagnosis												
1975–1979	6586	1.01	(0.98, 1.03)	5007	0.92	(0.90, 0.95)	257	0.88	(0.77, 0.99)	132	1.71	(1.43, 2.02)
1980–1984	6844	1.00	(0.98, 1.02)	5083	0.96	(0.93, 0.98)	457	0.94	(0.86, 1.03)	170	1.34	(1.15, 1.56)
1985–1989	7799	0.90	(0.88, 0.92)	5669	0.91	(0.88, 0.93)	354	0.91	(0.82, 1.01)	181	1.02	(0.88, 1.18)
1990–1994	7116	0.89	(0.87, 0.91)	4494	0.95	(0.92, 0.97)	421	0.93	(0.85, 1.03)	267	0.82	(0.72, 0.92)
1995–1999	5866	0.87	(0.85, 0.89)	2930	0.96	(0.93, 1.00)	382	0.98	(0.88, 1.08)	440	0.80	(0.73, 0.88)
2000–2004	3793	0.82	(0.80, 0.85)	1635	0.94	(0.89, 0.99)	313	0.95	(0.85, 1.06)	445	0.77	(0.70, 0.85)
2005–2009	1988	0.80	(0.76, 0.83)	829	0.95	(0.89, 1.02)	202	1.01	(0.87, 1.16)	277	0.83	(0.73, 0.93)
2010–2016	820	0.74	(0.69, 0.79)	326	0.89	(0.79, 0.99)	89	1.01	(0.81, 1.25)	127	0.76	(0.63, 0.90)
Coefficient, *p*_trend_	(− 0.009), *p* < 0.001			(+ 0.0003), *p* = 0.66			(+ 0.003), *p* = 0.11			(− 0.021), *p* < 0.001		

	Radiotherapy alone (*n* = 140,150)			Left-sided radiotherapy^a^ (*n* = 70,761)			Right-sided radiotherapy^a^ (*n* = 69,119)		
	O	SMR	95% CI	O	SMR	95% CI	O	SMR	95% CI
Calendar year of diagnosis									
1975–1979	1190	1.58	(1.49, 1.67)	672	1.77	(1.64, 1.91)	511	1.39	(1.27, 1.51)
1980–1984	1134	1.23	(1.16, 1.30)	650	1.37	(1.27, 1.48)	482	1.08	(0.99, 1.18)
1985–1989	1595	0.88	(0.84, 0.93)	841	0.93	(0.87, 0.99)	750	0.84	(0.78, 0.90)
1990–1994	1934	0.79	(0.75, 0.82)	973	0.78	(0.73, 0.83)	959	0.80	(0.75, 0.85)
1995–1999	2114	0.77	(0.73, 0.80)	1037	0.75	(0.70, 0.79)	1074	0.78	(0.74, 0.83)
2000–2004	1400	0.71	(0.67, 0.75)	709	0.72	(0.67, 0.77)	690	0.70	(0.65, 0.76)
2005–2009	680	0.63	(0.58, 0.67)	322	0.57	(0.51, 0.64)	357	0.68	(0.61, 0.75)
2010–2016	278	0.57	(0.50, 0.64)	142	0.57	(0.48, 0.68)	136	0.56	(0.47, 0.67)
Coefficient, *p*_trend_	(− 0.028), *p* < 0.001			(− 0.032), *p* < 0.001			(− 0.02), *p* < 0.001		

*O* observed heart disease events, *SMR* standardized mortality ratio, *CI* Confidence interval

^a^Restricted to women with unilateral disease and known laterality

The trends in heart disease SMRs for chemotherapy were similar in the sensitivity analysis with the inclusion of biologic modifiers to the chemotherapy category after 2013 (Supplemental Table A), but SMRs were slightly attenuated when censored at second cancers (Supplemental Table B).

#### By latency

The increasing heart disease SMRs among patients treated with chemotherapy alone and the decreasing SMRs among patients treated with radiotherapy alone and chemotherapy plus radiotherapy were also broadly similar by latency period (< 10, 10–19, 20 + years since diagnosis). Trends in heart disease SMRs were significantly increasing for patients treated with surgery only at 10 + years after diagnosis and increasing for chemotherapy alone at 10–19 years after diagnosis (Supplemental Table C).

#### By breast cancer stage

**Fig. 1:**
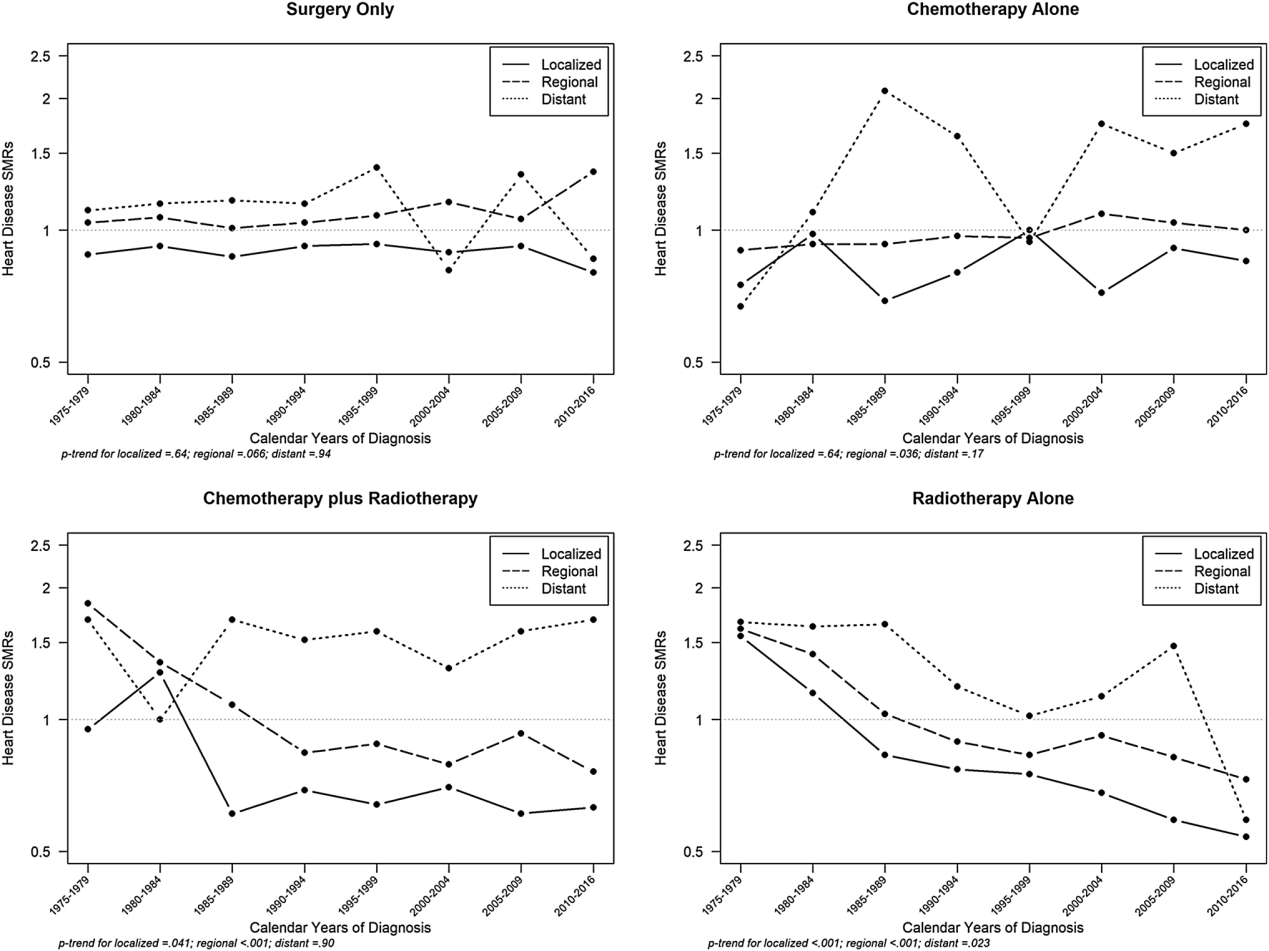
Trends in heart disease SMRs among breast cancer survivors by stage at cancer diagnosis and treatment type. *SMR* standardized mortality ratios

Declining trends in heart disease SMRs were evident among all stages for radiotherapy alone and among localized and regional cancers for chemotherapy plus radiotherapy (Fig. 1). Conversely, trends for heart disease mortality significantly increased among women diagnosed with regional stage breast cancer who received chemotherapy alone with an SMR of 0.90 (95%CI: 0.78, 1.04) from 1975–1979 to 1.00 (95%CI: 0.70, 1.37) in 2010–2016 (*p*_trend_ = 0.036).

#### By age at breast cancer diagnosis

**Fig. 2:**
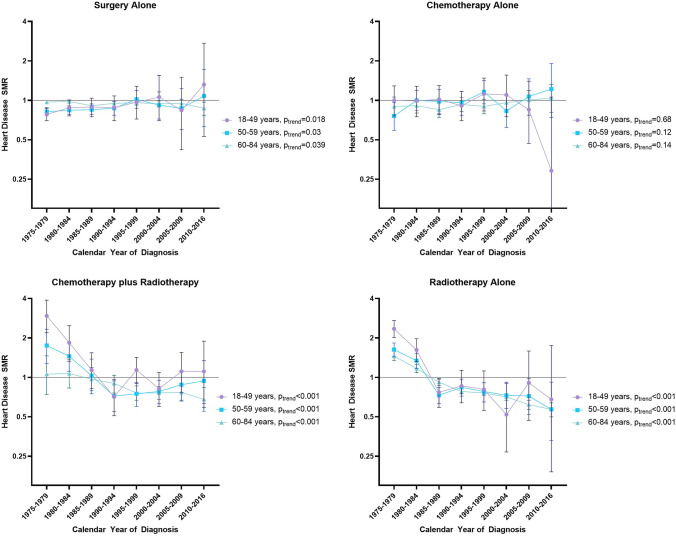
Heart disease SMRs by calendar year of diagnosis, age at diagnosis, and treatment type. *SMR* standardized mortality ratios

There were significant declining trends in SMRs for heart disease among all age groups of patients that received chemotherapy plus radiotherapy and radiotherapy alone (Fig. 2; Supplemental Table D). The greatest decline was observed among the youngest survivors (18–49 years at diagnosis) for women treated with chemotherapy plus radiotherapy (SMR of 2.95 [95%CI: 2.19, 3.89] from 1975–1979 to 1.11 [95%CI: 0.59, 1.89] in 2010–2016, *p*_trend_ < 0.001) and for women treated with radiotherapy alone (SMR of 2.35 [95%CI: 2.02, 2.72] declined to 0.68 [95%CI: 0.19, 1.75] for the same time period, *p*_trend_ < 0.001). For surgery only, there were increasing trends among women aged 18–59 and declining trends for older women.

### Cumulative mortality of heart disease

**Fig. 3:**
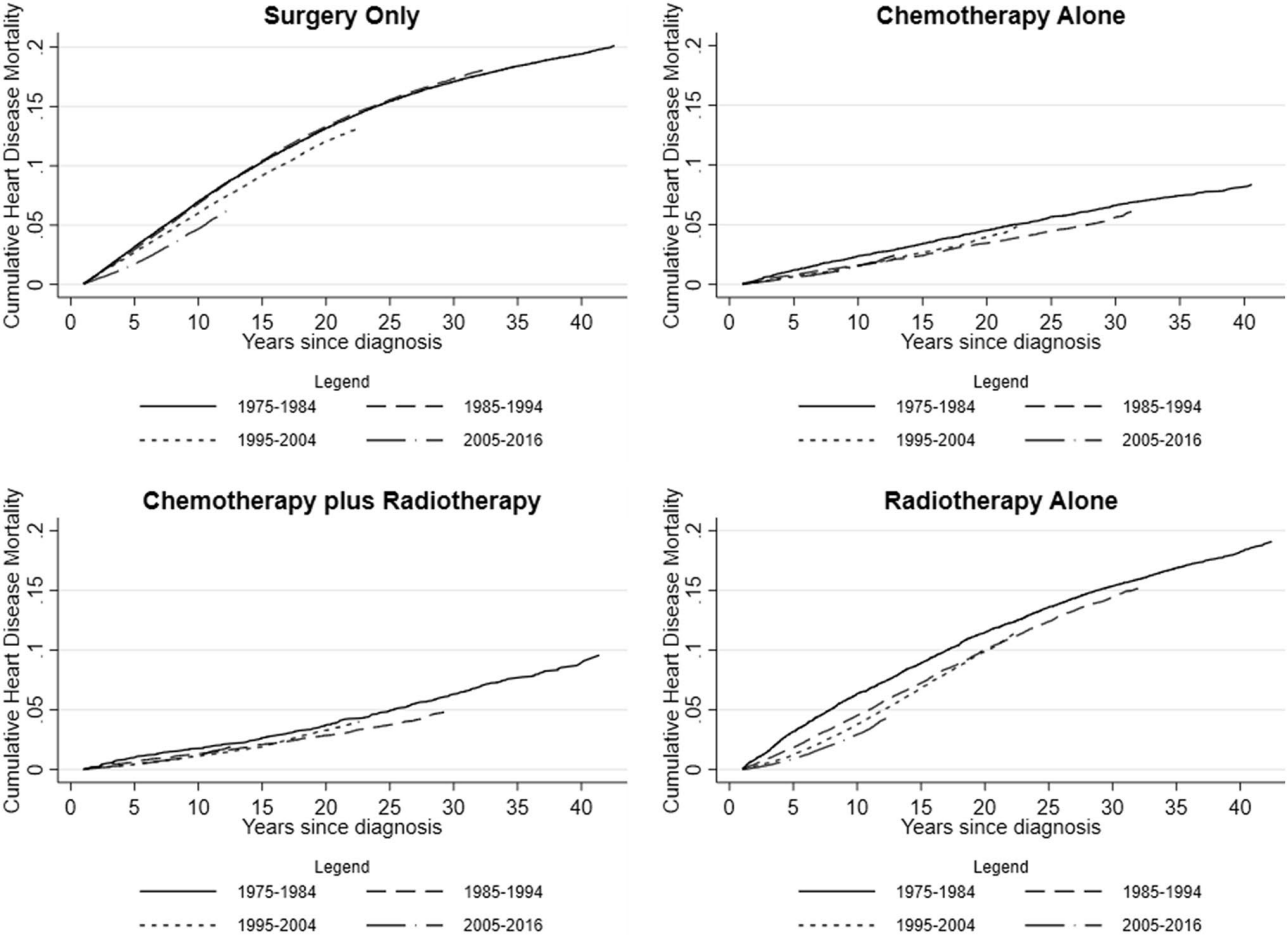
Cumulative mortality for heart disease among breast cancer survivors, by treatment type and calendar year of diagnosis

Cumulative mortality analyses demonstrated the varying clinical burden of heart disease by calendar year of breast cancer diagnosis and treatment type (Fig. 3). The 10-year cumulative mortality declined among survivors diagnosed from 1975–1984 to 2005–2016 treated with surgery only: 7.02% (95% CI: 6.80%, 7.23%) to 4.68% (95% CI: 4.39%, 4.99%), and with radiotherapy alone: 6.35% (95% CI: 5.95%, 6.77%) to 2.94% (95% CI: 2.73%, 3.16%). For the same time period, the declines in cumulative mortality of heart disease were smaller for the chemotherapy alone group: 2.38% (95% CI: 2.09%, 2.70%) to 1.58% (95% CI: 1.38%, 1.79%), and for the chemotherapy plus radiotherapy group: 1.78% (95% CI: 1.40%, 2.24%) to 1.21% (95% CI: 1.08%, 1.79%).

## Discussion

To our knowledge, this is the first descriptive study to provide a novel perspective on trends in heart disease mortality among US breast cancer survivors, accounting for heart disease mortality rates in the general population, by treatment regimen over 42 years of follow-up. We observed a significant declining trend in heart disease mortality among breast cancer survivors compared to the general population by calendar year of diagnosis, overall and for breast cancer survivors treated with radiotherapy alone. The greatest declines were observed among younger survivors treated with radiotherapy. In contrast, we observed an increasing trend in heart disease mortality for regional stage patients treated with chemotherapy alone.

Several studies have reported increased risk of death due to heart disease among US breast cancer survivors [8, 18–21]. Yet, to date, only two studies have described patterns of heart disease mortality among breast cancer survivors compared to the general population, and these did not evaluate the trends according to treatment group [22, 23]. Weberpals and colleagues studied patients treated more recently (2000–2011) and reported a lower risk of heart disease deaths among breast cancer survivors treated with chemotherapy or radiotherapy compared to the general population (SMR = 0.84 [95% 0.79, 0.90]) [23]. Our study adds to the literature by describing the long-term trends which demonstrates the likely impact of treatment changes with higher SMRs in the early years, especially between 1975 and 1984 when heart disease mortality was elevated compared to the general population. Sturgeon et al. assessed overall cardiovascular disease (CVD)—and specific CVD disease mortality among 28 cancer types diagnosed between 1973 and 2012. Patients diagnosed with breast cancer were among six cancer sites with a larger proportion of CVD deaths which increased by calendar year of diagnosis, but the study did not examine SMRs by treatment group [22].

Overall, breast cancer survivors in the US had a lower risk of heart disease mortality compared to the general population. This lower risk in heart disease SMRs may partially be explained by the healthy screenee bias [24], where some patients have heightened medical surveillance after breast cancer diagnosis and better healthcare access, and the increasing awareness of cardiovascular risk factors among healthcare providers and patients. Additionally, breast cancer survivors may be more likely to die of breast cancer in the first several years after diagnosis [18, 19], which we then accounted for in the cumulative mortality analyses. By examining stratified SMRs for heart disease, our study revealed differences by treatment type and elevated heart disease mortality compared to the general population in selected time periods.

Our finding of a declining trend in heart disease mortality among breast cancer survivors treated with radiotherapy is consistent with the changes in treatment practice. With increasing awareness of cardiotoxicity over time, as evidenced with development of clinical guidelines [7, 15, 25], there have been advances in cardioprotection strategies to minimize potential heart damage including positioning patients to displace heart during radiotherapy administration, more precise radiotherapy using imaging and brachytherapy, and alternative radiotherapy options (i.e., proton therapy) with potentially lower heart disease risk [14]. Because of proximity to the heart, left breast radiotherapy has a higher risk of heart disease compared to the right breast [10]. We observed a greater declining trend among left breast radiotherapy in the early years when radiotherapy techniques delivered higher doses to the heart. Modern radiotherapy techniques to reduce exposure to the heart likely contributed to similar heart disease SMRs by laterality after 1990. Results for the women treated with radiotherapy most recently should be interpreted cautiously because with shorter follow-up, cardiotoxicity may not yet be apparent.

The increase in heart disease mortality after chemotherapy alone among regional stage breast cancer survivors are likely driven by changing treatment patterns [11, 26], including increasing trastuzumab uptake. Trastuzumab received initial Federal Drug Administration (FDA) approval in 1998 and subsequent FDA approval for adjuvant treatment of HER2-positive node-positive breast cancers in 2006 [27]. HER2-positive breast cancers comprise of nearly 15% of breast cancer diagnoses [1], and the American Society of Clinical Oncology recommends trastuzumab for all women with HER2-positive node-positive cancers [28, 29]. Trastuzumab is associated with declines in cardiac function (measured via left ventricular ejection fraction) and when used sequentially with anthracyclines, the risk of heart disease is exponentially greater [6, 15, 20]. Trastuzumab without anthracyclines has not demonstrated long-term increased heart disease risk in early breast cancer clinical trials [30]. While there is evidence of declining anthracycline use in breast cancer treatment [11], this relatively recent change is unlikely to have impacted patterns of heart disease mortality. Additionally, changing patterns in staging definitions, cancer treatment regimens, use of neoadjuvant chemotherapy, and inclusion of gene expression panels (e.g., Oncotype DX) to assess recurrence and guide treatment decisions [1] can affect trends in heart disease mortality. These variables are unavailable in the SEER database or for the entire study period, but we attempted to account for time period changes by stratifying by year of breast cancer diagnosis. Specifically, we estimated cumulative mortality by calendar period of diagnosis to account for changes in treatments. Future research into detailed cancer treatment data and associations with cardiotoxicity are warranted to explore these trends.

Our results demonstrated women treated with surgery only who were aged 18–59 at breast cancer diagnosis have an increasing trend in heart disease compared to the general population but declining for women aged 60–84 years. The etiology is unclear for contrasting heart disease trends for the surgery only group by age. The surgery only treatment group largely consisted of older women, and advancing age is a shared risk factor for both breast cancer and heart disease [7]. Potential under-reported treatment (e.g., chemotherapy or radiotherapy) among the surgery only group may misclassify some women, as women diagnosed with advanced cancers may likely receive additional treatment beyond surgery.

A strength of this study is the large size of the population-based cohort and long-term follow-up. Our study has several limitations due to its registry-based design including lack of information on traditional cardiovascular disease risk factors (e.g., hypertension, diabetes, dyslipidemia), subsequent treatment, specific chemotherapy treatment agents, and potentially under-reported initial treatment. However, a previous report describe the sensitivity of chemotherapy and radiotherapy within SEER to be moderate (69% and 80%, respectively), and the positive predictive value high (91% and 98%, respectively) for breast cancer [31]. Further, we were not able to evaluate SMRs by breast cancer subtype (estrogen receptor, progesterone receptor, and human epidermal growth factor receptor 2 status) or by hormone therapy use since these data were not available consistently throughout our follow-up.

Characterizing heart disease mortality among breast cancer survivors is an important step in the context of cardio-oncology care and research. Breast cancer survivors are the largest group of cancer survivors, and maintaining optimal cardiovascular health is crucial as they are living many years post diagnosis and are at increased risk due to cancer treatment risk factors and mutual cancer and cardiovascular disease risk factors [1, 7, 21]. These mutual lifestyle and behavior risk factors include diet, obesity, physical activity, and smoking are associated with both the development of heart disease and breast cancer [7]. Independently, management of these risk factors can reduce breast cancer recurrence and prevent heart disease [32, 33]. While the SEER database lacks individual-level heart disease risk factors, the presented SMR analyses accounted for the declining heart disease mortality rates in the general population [34] reflecting changes in risk factors and treatment for heart disease. Studies and interventions to quantify and reduce risk factors for primordial heart disease prevention [33] among breast cancer survivors are an essential step.

Though we observed declining heart disease SMRs among breast cancer survivors treated with radiotherapy, it is important not to lose sight of this progress, as the association of heart disease still exists with radiotherapy use [8]. Clinical guidelines were developed for breast cancer survivors to recommend cardiovascular screening and assessment of cardiac function via cardiac magnetic resonance imaging (cMRI) or echocardiograms before treatment with anthracyclines, and before and during treatment when used sequentially with trastuzumab [7, 15, 25, 35]. Yet guidelines only recently included recommendations for cardiac screening of high risk patients who received anthracyclines within 1 year of completion of treatment and do not have long-term guidelines for cardiac screening [15]. Studies have demonstrated late effects of cardiotoxicity from cancer treatment [18], in line with our increasing heart disease SMRs among regional stage breast cancer patients who received chemotherapy alone. Research detailing consequences of late occurring cardiotoxicity and the effects of screening on long-term reduction of heart disease risk are lacking.

In addition to screening recommendations, recent studies and current trials are examining the effectiveness of pharmacological cardiotoxicity prevention using angiotensin-converting enzyme (ACE) inhibitors, beta blockers, and statins, which can protect against declines in cardiac function [36, 37]. Their common use in the general population, combined with their potential for safe and effective use with known cardiotoxic treatment, may allow for cardiac protection while not compromising breast cancer survival. With the available data, clinical implications from this study further support inclusion of cardiovascular management and discussions of potential cardiotoxicity before, during, and throughout cancer survivorship. Clinical care should be individualized to the patient; however, it is essential to provide patient care concordant with clinical recommendations to ensure appropriate cardiovascular screenings are obtained (e.g., cMRI and echocardiograms) for breast cancer survivors receiving cardiotoxic cancer treatment.

In conclusion, we found that heart disease mortality among US breast cancer survivors has declined compared to the general population, overall and among patients treated with radiotherapy, but has increased among regional stage patients treated with chemotherapy. Further work is needed to understand specific chemotherapy agents that contribute to heart disease and to reduce the heart disease burden among breast cancer survivors.

## Supplementary Information

Below is the link to the electronic supplementary material.

Supplementary file1 (DOCX 58 KB)

## Data Availability

The datasets analyzed during the current study are available from the SEER registry https://seer.cancer.gov/.

## Code availability

Available upon request.

## Declarations

### Disclaimer

The funder had no role in the design of the study; the collection, analysis, or interpretation of the data; the writing of the manuscript; or the decision to submit the manuscript for publication. The results reported here and the conclusions derived are the sole responsibility of the authors. The opinions expressed by the authors are their own and this material should not be interpreted as representing the official viewpoint of the U.S. Department of Health and Human Services, the National Institutes of Health or the National Cancer Institute.
