# Supplementary material for: Trends in heart disease mortality among breast cancer survivors in the US, 1975–2017
Source: Breast Cancer Res Treat. 2022 Feb 2;192(3):611–22. doi: 10.1007/s10549-022-06515-5 (PMC8960573; doi:10.1007/s10549-022-06515-5)
Supplement: Supplementary file 1 — Supplementary file1 (DOCX 58 KB) [file 10549_2022_6515_MOESM1_ESM.docx]

**Supplement A.**

For breast cancer stage, we used a combination of SEER Historic Stage A (1973-2015) for breast cancer patients diagnosed between 1975 and 2015, and we used SEER Combined Summary Stage (2004+) for patients diagnosed in 2016. Localized breast cancer includes cancer confined to breast tissue and fat including nipple and/or areola and Paget disease with or without underlying tumor. Regional stage breast cancer included cancer of the breast by direct extension, with lymph nodes, or both. Distant stage included metastasis to other sites or distant lymph nodes, not otherwise specified. Detailed description of SEER’s staging variables can be found here: <https://seer.cancer.gov/tools/ssm/2018-Summary-Stage-Manual.pdf> Additionally, the SEER program strives to make Localized/Regional/Distant stage variables consistent across years as described here: <https://seer.cancer.gov/seerstat/variables/seer/lrd-stage/>

**Supplemental Table A.** Heart disease SMRs by calendar year of diagnosis and treatment type, after inclusion of biologic modifiers to the chemotherapy category after 2013.

|  | Overall  (N = 516,977) | | | Surgery Only (n = 197,319) | | | | Chemotherapy Alone (n = 76,670) | | | | | | | Chemotherapy plus Radiotherapy  (n = 103,019) | | | | | |  |
| --- | --- | --- | --- | --- | --- | --- | --- | --- | --- | --- | --- | --- | --- | --- | --- | --- | --- | --- | --- | --- | --- |
|  | O | SMR | 95% CI | O | SMR | 95% CI | O | | SMR | | 95% CI | | O | | | SMR | | 95% CI | |  |  |
| Calendar Year of Diagnosis |  |  |  |  |  |  |  | |  | |  | |  | | |  | |  | |  |  |
| 1975-1979 | 6,586 | 1.01 | (0.98,1.03) | 5,007 | 0.92 | (0.90,0.95) | 257 | | 0.88 | | (0.77, 0.99) | | 132 | | | 1.71 | | (1.43,2.02) | |  |  |
| 1980-1984 | 6,844 | 1.00 | (0.98,1.02) | 5,083 | 0.96 | (0.93,0.98) | 457 | | 0.94 | | (0.86, 1.03) | | 170 | | | 1.34 | | (1.15,1.56) | |  |  |
| 1985-1989 | 7,799 | 0.90 | (0.88,0.92) | 5,669 | 0.91 | (0.88,0.93) | 354 | | 0.91 | | (0.82, 1.01) | | 181 | | | 1.02 | | (0.88,1.18) | |  |  |
| 1990-1994 | 7,116 | 0.89 | (0.87,0.91) | 4,494 | 0.95 | (0.92,0.97) | 421 | | 0.93 | | (0.85, 1.03) | | 267 | | | 0.82 | | (0.72,0.92) | |  |  |
| 1995-1999 | 5,866 | 0.87 | (0.85,0.89) | 2,930 | 0.96 | (0.93,1.00) | 382 | | 0.98 | | (0.88, 1.08) | | 440 | | | 0.80 | | (0.73,0.88) | |  |  |
| 2000-2004 | 3,793 | 0.82 | (0.80,0.85) | 1,635 | 0.94 | (0.89,0.99) | 313 | | 0.95 | | (0.85, 1.06) | | 445 | | | 0.77 | | (0.70,0.85) | |  |  |
| 2005-2009 | 1,988 | 0.80 | (0.76,0.83) | 829 | 0.95 | (0.89,1.02) | 202 | | 1.01 | | (0.87, 1.16) | | 277 | | | 0.83 | | (0.73,0.93) | |  |  |
| 2010-2016 | 820 | 0.74 | (0.69,0.79) | 325 | 0.88 | (0.79,0.99) | 90 | | 1.01 | | (0.81, 1.24) | | 128 | | | 0.76 | | (0.63,0.90) | |  |  |
| coefficient,  p_trend_ | (-0.009), p<.0001 | | | (+0.0003), p=0.37 | | | (+0.003), p=0.11 | | | | | | (-0.021), p<0.001 | | | | | | |  |  |
|  | Radiotherapy Alone (n = 139,969) | | | Left-sided Radiotherapy^a^  (n = 70,665) | | | Right-sided Radiotherapy^a^  (n = 69,034) | | | | | |  | | |  | |  | |  |  |
|  | O | SMR | 95% CI | O | SMR | 95% CI | O | | SMR | | 95% CI | |  | | |  | |  | |  |  |
| Calendar Year of Diagnosis |  |  |  |  |  |  |  | |  | |  | |  | | |  | |  | |  |  |
| 1975-1979 | 1,190 | 1.58 | (1.49, 1.67) | 672 | 1.77 | (1.64,1.91) | 511 | | | 1.39 | | (1.27,1.51) | |  | | |  | |  | | |
| 1980-1984 | 1,134 | 1.23 | (1.16, 1.30) | 650 | 1.37 | (1.27.1.48) | 482 | | | 1.08 | | (0.99,1.18) | |  | | |  | |  | | |
| 1985-1989 | 1,595 | 0.88 | (0.84, 0.93) | 841 | 0.93 | (0.87,0.99) | 750 | | | 0.84 | | (0.78,0.90) | |  | | |  | |  | | |
| 1990-1994 | 1,934 | 0.79 | (0.75, 0.82) | 973 | 0.78 | (0.73,0.83) | 959 | | | 0.80 | | (0.75,0.85) | |  | | |  | |  | | |
| 1995-1999 | 2,114 | 0.77 | (0.73, 0.80) | 1,037 | 0.75 | (0.70,0.79) | 1,074 | | | 0.78 | | (0.74,0.83) | |  | | |  | |  | | |
| 2000-2004 | 1,400 | 0.71 | (0.67, 0.75) | 709 | 0.72 | (0.67,0.77) | 690 | | | 0.70 | | (0.65,0.76) | |  | | |  | |  | | |
| 2005-2009 | 680 | 0.63 | (0.58, 0.67) | 322 | 0.57 | (0.51,0.64) | 357 | | | 0.68 | | (0.61,0.75) | |  | | |  | |  | | |
| 2010-2016 | 277 | 0.57 | (0.50, 0.64) | 141 | 0.57 | (0.48,0.68) | 136 | | | 0.56 | | (0.47,0.67) | |  | | |  | |  | | |
| coefficient,  p_trend_ | (-0.028), p<0.001 | | | (-0.034), p<0.001 | | | (-0.022), p<0.001 | | | | | |  | | |  | |  | |  |  |

Note. O=Observed heart disease events. SMR=Standardized mortality ratio. CI=Confidence interval. Chemotherapy group included the biologic therapy/immunotherapy category in the chemotherapy group for breast cancer diagnoses after 2013. ^a^Restricted to women with unilateral disease and known laterality.

**Supplement Table B.** Heart disease SMRs by calendar year of diagnosis and treatment type, censored at second invasive cancer.

|  | Overall  (N = 501,628) | | | Surgery Only (n = 190,727) | | | | Chemotherapy Alone (n = 73,633) | | | Chemotherapy plus Radiotherapy  (n = 100,182) | | | | |  |  |  |  |
| --- | --- | --- | --- | --- | --- | --- | --- | --- | --- | --- | --- | --- | --- | --- | --- | --- | --- | --- | --- |
|  | O | SMR | 95% CI | O | SMR | 95% CI | O | | SMR | 95% CI | | O | SMR | 95% CI |  |  |  |  |  |
| Calendar Year of Diagnosis |  |  |  |  |  |  |  | |  |  | |  |  |  |  |  |  |  |  |
| 1975-1979 | 5,558 | 0.99 | (0.96,1.01) | 4,231 | 0.91 | (0.88,0.93) | 220 | | 0.91 | (0.79,1.03) | | 114 | 1.69 | (1.40,2.04) |  |  |  |  |  |
| 1980-1984 | 5,776 | 0.99 | (0.96,1.01) | 4,330 | 0.95 | (0.92,0.98) | 370 | | 0.90 | (0.81,1.00) | | 134 | 1.31 | (1.09,1.55) |  |  |  |  |  |
| 1985-1989 | 6,482 | 0.88 | (0.86,0.90) | 4,740 | 0.89 | (0.86,0.91) | 287 | | 0.87 | (0.77,0.98) | | 148 | 1.00 | (0.84,1.17) |  |  |  |  |  |
| 1990-1994 | 6,032 | 0.88 | (0.85,0.90) | 3,855 | 0.93 | (0.90,0.96) | 339 | | 0.87 | (0.78,0.97) | | 219 | 0.79 | (0.69,0.90) |  |  |  |  |  |
| 1995-1999 | 5,043 | 0.85 | (0.83,0.88) | 2,557 | 0.96 | (0.92,0.99) | 306 | | 0.91 | (0.81,1.02) | | 375 | 0.79 | (0.71,0.87) |  |  |  |  |  |
| 2000-2004 | 3,342 | 0.81 | (0.78,0.84) | 1,464 | 0.94 | (0.89,0.99) | 280 | | 0.95 | (0.84,1.07) | | 378 | 0.73 | (0.66,0.81) |  |  |  |  |  |
| 2005-2009 | 1,764 | 0.78 | (0.74,0.81) | 741 | 0.94 | (0.87,1.01) | 179 | | 0.99 | (0.85,1.15) | | 241 | 0.78 | (0.69,0.89) |  |  |  |  |  |
| 2010-2016 | 752 | 0.73 | (0.68,0.78) | 296 | 0.87 | (0.77,0.97) | 79 | | 0.99 | (0.79,1.24) | | 121 | 0.77 | (0.64,0.92) |  |  |  |  |  |
| coefficient,  p_trend_ | (-0.009), p<0.001 | | | (+0.0007), p=0.37 | | | (+0.003), p=0.22 | | | | | (-0.021), p<0.001 | | |  | |  |  |  |
|  | Radiotherapy Alone  (n = 137,086) | | | Left-sided Radiotherapy^a^  (n = 69,296) | | | Right-sided Radiotherapy^a^  (n = 67,528) | | | | |  |  |  |  |  |  |  |  |
|  | O | SMR | 95% CI | O | SMR | 95% CI | O | | SMR | 95% CI | |  |  |  |  |  |  |  |  |
| Calendar Year of Diagnosis |  |  |  |  |  |  |  | |  |  | |  |  |  |  |  |  |  |  |
| 1975-1979 | 993 | 1.52 | (1.43,1.62) | 562 | 1.72 | (1.59,1.87) | 425 | | 1.32 | (1.20,1.46) | |  |  |  |  |  |  |  |  |
| 1980-1984 | 942 | 1.23 | (1.15,1.31) | 531 | 1.36 | (1.25,1.48) | 409 | | 1.09 | (0.99,1.20) | |  |  |  |  |  |  |  |  |
| 1985-1989 | 1,307 | 0.87 | (0.82,0.91) | 682 | 0.90 | (0.84,0.97) | 622 | | 0.83 | (0.76,0.89) | |  |  |  |  |  |  |  |  |
| 1990-1994 | 1,619 | 0.78 | (0.74,0.82) | 802 | 0.76 | (0.71,0.81) | 815 | | 0.80 | (0.74,0.85) | |  |  |  |  |  |  |  |  |
| 1995-1999 | 1,805 | 0.75 | (0.71,0.78) | 884 | 0.73 | (0.68,0.78) | 918 | | 0.76 | (0.71,0.81) | |  |  |  |  |  |  |  |  |
| 2000-2004 | 1,220 | 0.69 | (0.65,0.73) | 619 | 0.70 | (0.64,0.76) | 600 | | 0.68 | (0.63,0.74) | |  |  |  |  |  |  |  |  |
| 2005-2009 | 603 | 0.61 | (0.56,0.66) | 291 | 0.57 | (0.50,0.64) | 311 | | 0.65 | (0.58,0.72) | |  |  |  |  |  |  |  |  |
| 2010-2016 | 256 | 0.56 | (0.49,0.63) | 135 | 0.58 | (0.49,0.69) | 121 | | 0.54 | (0.45,0.64) | |  |  |  |  |  |  |  |  |
| coefficient,  p_trend_ | (-0.028), p<0.001 | | | (-0.031), p<0.001 | | | (-0.021), p<0.001 | | | | |  |  |  |  |  |  |  |  |

Note. O=Observed heart disease events. SMR=Standardized mortality ratio. CI=Confidence interval. ^a^Restricted to women with unilateral disease and known laterality.

**Supplement Table C.** Heart disease SMRs by calendar year of diagnosis, treatment type, and latency period.

|  | Surgery Only (n = 197,449) | | | | | | | | | Chemotherapy Alone (n = 76,479) | | | | | | | | |
| --- | --- | --- | --- | --- | --- | --- | --- | --- | --- | --- | --- | --- | --- | --- | --- | --- | --- | --- |
|  | <10 years | | | 10-19 years | | | 20+ years | | | <10 years | | | 10-19 years | | | 20+ years | | |
|  | O | SMR | 95% CI | O | SMR | 95% CI | O | SMR | 95% CI | O | SMR | 95% CI | O | SMR | 95% CI | O | SMR | 95% CI |
| Calendar Year of Diagnosis |  |  |  |  |  |  |  |  |  |  |  |  |  |  |  |  |  |  |
| 1975-1979 | 1764 | 0.94 | (0.90,0.98) | 1579 | 0.93 | (0.88,0.98) | 1,664 | 0.90 | (0.86,0.95) | 83 | 0.96 | (0.77,1.19) | 66 | 0.86 | (0.66,1.09) | 108 | 0.83 | (0.68,1.00) |
| 1980-1984 | 1949 | 0.94 | (0.90,0.99) | 1719 | 0.95 | (0.90,0.99) | 1,415 | 0.99 | (0.94,1.05) | 138 | 0.93 | (0.78,1.10) | 135 | 0.86 | (0.72,1.02) | 184 | 1.02 | (0.88,1.18) |
| 1985-1989 | 2157 | 0.83 | (0.79,0.86) | 2172 | 0.93 | (0.89,0.97) | 1,340 | 1.03 | (0.97,1.08) | 115 | 0.96 | (0.79,1.15) | 116 | 0.84 | (0.69,1.01) | 123 | 0.94 | (0.78,1.12) |
| 1990-1994 | 1999 | 0.88 | (0.84,0.92) | 1818 | 0.98 | (0.93,1.02) | 677 | 1.11 | (1.02,1.19) | 139 | 0.92 | (0.77,1.08) | 177 | 0.94 | (0.81,1.09) | 105 | 0.94 | (0.77,1.14) |
| 1995-1999 | 1482 | 0.88 | (0.84,0.93) | 1361 | 1.06 | (1.01,1.12) | 87 | 0.98 | (0.79,1.21) | 157 | 0.9 | (0.77,1.06) | 199 | 1.01 | (0.87,1.16) | 26 | 1.39 | (0.91,2.03) |
| 2000-2004 | 1016 | 0.88 | (0.83,0.93) | 619 | 1.06 | (0.98,1.14) |  |  |  | 165 | 0.88 | (0.75,1.03) | 148 | 1.04 | (0.88,1.22) |  |  |  |
| 2005-2009 | 739 | 0.93 | (0.86,1.00) | 90 | 1.15 | (0.92,1.41) |  |  |  | 176 | 0.99 | (0.85,1.14) | 26 | 1.17 | (0.77,1.72) |  |  |  |
| 2010-2016 | 326 | 0.89 | (0.79,0.99) |  |  |  |  |  |  | 89 | 1.01 | (0.81,1.25) |  |  |  |  |  |  |
| coefficient,  p_trend_ | (-0.002), p=0.12 | | | (+0.006), p<0.001 | | | (+0.011), p<0.001 | | | (+0.0005), p=0.87 | | | (+0.011), p=0.011 | | | (+0.01), p=0.15 | | |
|  | Chemotherapy plus Radiotherapy  (n = 102,838) | | | | | | | | | Radiotherapy Alone (n = 140,150) | | | | | | | | |
|  | <10 years | | | 10-19 years | | | 20+ years | | | <10 years | | | 10-19 years | | | 20+ years | | |
|  | O | SMR | 95% CI | O | SMR | 95% CI | O | SMR | 95% CI | O | SMR | 95% CI | O | SMR | 95% CI | O | SMR | 95% CI |
| Calendar Year of Diagnosis |  |  |  |  |  |  |  |  |  |  |  |  |  |  |  |  |  |  |
| 1975-1979 | 25 | 1.04 | (0.67,1.53) | 32 | 1.54 | (1.05,2.17) | 75 | 2.31 | (1.82,2.90) | 450 | 1.57 | (1.43,1.72) | 328 | 1.44 | (1.29,1.61) | 412 | 1.73 | (1.56,1.90) |
| 1980-1984 | 42 | 1.08 | (0.78,1.46) | 42 | 1.13 | (0.81,1.53) | 86 | 1.69 | (1.35,2.09) | 395 | 1.12 | (1.02,1.24) | 361 | 1.18 | (1.07,1.31) | 378 | 1.42 | (1.28,1.57) |
| 1985-1989 | 52 | 0.94 | (0.70,1.23) | 64 | 1.07 | (0.82,1.36) | 65 | 1.05 | (0.81,1.34) | 516 | 0.83 | (0.76,0.91) | 586 | 0.84 | (0.78,0.91) | 493 | 1.01 | (0.92,1.10) |
| 1990-1994 | 89 | 0.82 | (0.66,1.01) | 112 | 0.81 | (0.67,0.98) | 66 | 0.83 | (0.64,1.06) | 674 | 0.69 | (0.64,0.75) | 865 | 0.81 | (0.76,0.87) | 395 | 0.95 | (0.86,1.05) |
| 1995-1999 | 168 | 0.73 | (0.63,0.85) | 251 | 0.84 | (0.74,0.95) | 21 | 1.00 | (0.62,1.53) | 818 | 0.63 | (0.58,0.67) | 1,191 | 0.88 | (0.83,0.93) | 105 | 1.06 | (0.87,1.28) |
| 2000-2004 | 216 | 0.68 | (0.59,0.77) | 229 | 0.9 | (0.78,1.02) |  |  |  | 728 | 0.61 | (0.57,0.66) | 672 | 0.86 | (0.80,0.93) |  |  |  |
| 2005-2009 | 228 | 0.77 | (0.67,0.88) | 49 | 1.23 | (0.91,1.62) |  |  |  | 578 | 0.6 | (0.55,0.65) | 102 | 0.84 | (0.68,1.01) |  |  |  |
| 2010-2016 | 127 | 0.76 | (0.63,0.90) |  |  |  |  |  |  | 278 | 0.57 | (0.50,0.64) |  |  |  |  |  |  |
| coefficient,  p_trend_ | (-0.01), p=0.011 | | | (-0.011), p=0.034 | | | (-0.06), p<0.001 | | | (-0.027), p<0.001 | | | (-0.015), p<.001 | | | (-0.037), p<0.001 | | |

Note. O=Observed heart disease events. SMR=Standardized mortality ratio. CI=Confidence interval.

**Supplement Table D.** Heart disease SMRs by calendar year of diagnosis, age at diagnosis, and treatment type.

|  | Surgery Only (n = 197,449) | | | | | | | | | Chemotherapy Alone (n = 76,479) | | | | | | | | |
| --- | --- | --- | --- | --- | --- | --- | --- | --- | --- | --- | --- | --- | --- | --- | --- | --- | --- | --- |
|  | 18-49 years | | | 50-59 years | | | 60-84 years | | | 18-49 years | | | 50-59 years | | | 60-84 years | | |
|  | O | SMR | 95% CI | O | SMR | 95% CI | O | SMR | 95% CI | O | SMR | 95% CI | O | SMR | 95% CI | O | SMR | 95% CI |
| Calendar Year of Diagnosis |  |  |  |  |  |  |  |  |  |  |  |  |  |  |  |  |  |  |
| 1975-1979 | 363 | 0.78 | (0.70,0.87) | 949 | 0.82 | (0.77,0.88) | 3695 | 0.97 | (0.94,1.00) | 59 | 1.00 | (0.76,1.29) | 67 | 0.76 | (0.59,0.96) | 131 | 0.90 | (0.75,1.06) |
| 1980-1984 | 217 | 0.88 | (0.76,1.00) | 698 | 0.84 | (0.78,0.91) | 4168 | 0.98 | (0.95,1.01) | 57 | 0.99 | (0.75,1.28) | 128 | 1.00 | (0.84,1.19) | 272 | 0.91 | (0.80,1.02) |
| 1985-1989 | 152 | 0.89 | (0.75,1.04) | 508 | 0.85 | (0.78,0.92) | 5009 | 0.91 | (0.89,0.94) | 63 | 1.02 | (0.78,1.30) | 86 | 0.98 | (0.79,1.21) | 205 | 0.85 | (0.74,0.98) |
| 1990-1994 | 87 | 0.88 | (0.70,1.08) | 257 | 0.87 | (0.77,0.98) | 4150 | 0.95 | (0.92,0.98) | 63 | 0.91 | (0.07,1.17) | 94 | 0.96 | (0.77,1.17) | 264 | 0.93 | (0.82,1.05) |
| 1995-1999 | 50 | 0.97 | (0.72,1.28) | 159 | 1.02 | (0.87,1.19) | 2721 | 0.96 | (0.92,0.99) | 49 | 1.12 | (0.83,1.48) | 95 | 1.16 | (0.94,1.42) | 238 | 0.9 | (0.79,1.02) |
| 2000-2004 | 27 | 1.06 | (0.70,1.55) | 74 | 0.92 | (0.72,1.16) | 1534 | 0.94 | (0.89,0.99) | 31 | 1.10 | (0.75,1.56) | 49 | 0.83 | (0.62,1.10) | 233 | 0.96 | (0.84,1.09) |
| 2005-2009 | 11 | 0.84 | (0.42,1.50) | 32 | 0.87 | (0.60,1.23) | 786 | 0.95 | (0.89,1.02) | 15 | 0.85 | (0.47,1.40) | 40 | 1.07 | (0.77,1.46) | 147 | 1.01 | (0.85,1.19) |
| 2010-2016 | 7 | 1.32 | (0.53,2.73) | 17 | 1.08 | (0.63,1.72) | 302 | 0.87 | (0.77,0.97) | 2 | 0.29 | (0.04,1.06) | 19 | 1.22 | (0.74,1.91) | 68 | 1.04 | (0.81,1.32) |
| coefficient,  p_trend_ |  | (+0.01), p=0.018 | |  | (+0.006), p=0.03 | |  | (-0.002), p=0.039 | |  | (-0.002), p=0.68 | |  | (+0.007), p=0.12 | |  | (+0.004), p=0.14 | |
|  | Chemotherapy plus Radiotherapy  (n = 102,838) | | | | | | | | | Radiotherapy Alone (n = 140,150) | | | | | | | | |
|  | 18-49 years | | | 50-59 years | | | 60-84 years | | | 18-49 years | | | 50-59 years | | | 60-84 years | | |
|  | O | SMR | 95% CI | O | SMR | 95% CI | O | SMR | 95% CI | O | SMR | 95% CI | O | SMR | 95% CI | O | SMR | 95% CI |
| Calendar Year of Diagnosis |  |  |  |  |  |  |  |  |  |  |  |  |  |  |  |  |  |  |
| 1975-1979 | 50 | 2.95 | (2.19,3.89) | 46 | 1.75 | (1.28,2.33) | 36 | 1.06 | (0.74,1.46) | 178 | 2.35 | (2.02,2.72) | 292 | 1.63 | (1.45,1.83) | 720 | 1.45 | (1.34,1.56) |
| 1980-1984 | 43 | 1.84 | (1.33,2.48) | 61 | 1.45 | (1.11,1.86) | 66 | 1.07 | (0.83,1.37) | 100 | 1.62 | (1.32,1.97) | 226 | 1.34 | (1.17,1.52) | 808 | 1.17 | (1.09,1.25) |
| 1985-1989 | 42 | 1.14 | (0.82,1.54) | 45 | 1.03 | (0.75,1.38) | 94 | 0.97 | (0.79,1.19) | 62 | 0.77 | (0.59,0.98) | 183 | 0.73 | (0.63,0.84) | 1350 | 0.92 | (0.87,0.97) |
| 1990-1994 | 43 | 0.71 | (0.51,0.96) | 58 | 0.72 | (0.55,0.94) | 166 | 0.90 | (0.76,1.04) | 50 | 0.86 | (0.64,1.13) | 174 | 0.84 | (0.72,0.97) | 1710 | 0.78 | (0.75,0.82) |
| 1995-1999 | 79 | 1.14 | (0.90,1.42) | 93 | 0.75 | (0.60,0.91) | 268 | 0.76 | (0.67,0.86) | 35 | 0.81 | (0.56,1.12) | 143 | 0.78 | (0.65,0.91) | 1936 | 0.76 | (0.73,0.80) |
| 2000-2004 | 47 | 0.82 | (0.60,1.09) | 97 | 0.78 | (0.63,0.95) | 301 | 0.76 | (0.68,0.85) | 12 | 0.52 | (0.27,0.92) | 80 | 0.73 | (0.58,0.90) | 1308 | 0.71 | (0.67,0.75) |
| 2005-2009 | 34 | 1.11 | (0.77,1.55) | 61 | 0.88 | (0.67,1.13) | 182 | 0.77 | (0.66,0.89) | 12 | 0.91 | (0.47,1.59) | 41 | 0.72 | (0.52,0.98) | 627 | 0.62 | (0.57,0.67) |
| 2010-2016 | 13 | 1.11 | (0.59,1.89) | 29 | 0.94 | (0.63,1.35) | 85 | 0.68 | (0.55,0.84) | 4 | 0.68 | (0.19,1.75) | 16 | 0.57 | (0.33,0.92) | 258 | 0.57 | (0.50,0.64) |
| coefficient,  p_trend_ |  | (-0.032), p<0.001 | |  | (-0.021), p<0.001 | |  | (-0.014), p<0.001 | |  | (-0.055), p<0.001 | |  | (-0.033), p<0.001 | |  | (-0.025), p<0.001 | |

Note. O=Observed heart disease events. SMR=Standardized mortality ratio. CI=Confidence interval.
